# Acute Effects of Strength and Endurance Training on Bone Turnover Markers in Young Adults and Elderly Men

**DOI:** 10.3389/fendo.2022.915241

**Published:** 2022-06-30

**Authors:** Astrid Kamilla Stunes, Cathrine Langlie Brobakken, Md Abu Jafar Sujan, Norun Aagård, Martin Siksjø Brevig, Eivind Wang, Unni Syversen, Mats Peder Mosti

**Affiliations:** ^1^ Department of Clinical and Molecular Medicine, Faculty of Medicine and Health Sciences, Norwegian University of Science and Technology, Trondheim, Norway; ^2^ Medical Clinic, St. Olavs University Hospital, Trondheim, Norway; ^3^ Department of Circulation and Medical Imaging, Faculty of Medicine and Health Sciences, Norwegian University of Science and Technology, Trondheim, Norway; ^4^ Myworkout, Medical Rehabilitation Clinic, Trondheim, Norway; ^5^ Faculty of Health and Social Sciences, Molde University College, Molde, Norway; ^6^ Department of Psychosis and Rehabilitation, Psychiatry Clinic, St. Olavs University Hospital, Trondheim, Norway; ^7^ Department of Endocrinology, St. Olavs University Hospital, Trondheim, Norway; ^8^ Department of Research and Development, Clinic of Substance Use and Addiction Medicine, St. Olavs University Hospital, Trondheim, Norway

**Keywords:** exercise, sclerostin, lipocalin-2, HIIT (high-intensity interval training), strength training, CTX-1, P1NP, osteocalcin

## Abstract

**Context:**

Exercise is recognized as an important strategy to prevent bone loss, but its acute effects on bone turnover markers (BTMs) and related markers remain uncertain.

**Objective:**

To assess the acute effects of two different exercise modes on BTMs and related markers in young adults of both sexes and elderly men.

**Design, Setting, Participants:**

This was a three-group crossover within-subjects design study with a total of 53 participants—19 young women (aged 22–30), 20 young men (aged 21–30 years), and 14 elderly men (aged 63–74 years)—performing two different exercise sessions [strength training (ST) and high-intensity interval training (HIIT)] separated by 2 weeks, in a supervised laboratory setting.

**Main Outcome Measures:**

Plasma volume-corrected serum measurements of the BTMs C-terminal telopeptide of type 1 collagen (CTX-I) and procollagen of type 1 N-terminal propeptide (P1NP), total osteocalcin (OC), sclerostin, and lipocalin-2 (LCN2) at baseline, immediately after, and 3 and 24 h after each of the two exercise modes were performed.

**Results and Conclusion:**

Analyses revealed sex- and age-dependent differences in BTMs and related bone markers at baseline and time-, sex-, and age-dependent differences in response to exercise. No differences between exercise modes were observed for BTM response except for sclerostin in young men and LCN2 in elderly men. An acute, transient, and uniform increase in P1NP/CTX-1 ratio was found in young participants, demonstrating that beneficial skeletal effects on bone metabolism can be attained through both aerobic endurance and resistance exercise, although this effect seems to be attenuated with age. The acute effects of exercise on bone-related biomarkers were generally blunted after 24 h, suggesting that persistent alterations following prolonged exercise interventions should be assessed at later time points.

## Introduction

Already in 1638, Galileo Galilei stated that “loading is required to preserve bone mass” (1). In the 1980s, the “mechanostat” theory, which describes the mechanism underlying the load-induced bone adaptation, was introduced by Harold Frost, who also identified the osteocyte as the “mechanostat” of bone (2, 3). Mechanical load causes dynamic fluid shifts in bone canalicular networks, which are sensed by bone-embedded osteocytes (4). The osteocytes convert the mechanical signal into biochemical signals transmitted to the effector cells, osteoclasts, and osteoblasts, impacting bone turnover (4).

Mechanical load can improve bone health and reduce the risk of osteoporosis; hence, physical activity and exercise are now regarded as beneficial interventions to prevent bone loss in the elderly (5) as well as in young subjects (6). In children, exercise/mechanical load is necessary to obtain an optimal peak bone mass to prevent future osteoporosis and fractures (7, 8). In the context of musculoskeletal health, strength training (ST) is widely advocated as a countermeasure for age- and lifestyle-related declines (9, 10). We have previously shown that maximal ST (MST) in hack squat provides a simple, low-volume training method to improve skeletal properties and neuromuscular function in young adults and postmenopausal women (11–13). Hack squat MST is a lower-extremity exercise that provides compressive load through the spine and the hip, which are sites particularly prone to osteoporotic fractures. Aerobic high-intensity interval training (HIIT) is a well-recognized intervention for cardiovascular and metabolic health that has become widely advocated in the last few years (14–16). HIIT is often performed as treadmill running, which has been shown to improve bone metabolism and bone mineral density (BMD), both acutely and long term (17, 18).

Most studies state a positive relationship between BMD and exercise in adults and the elderly and that exercise contributes to maintaining BMD and/or reducing bone loss (19). Conflicting results regarding the effects of training, as well as the load, intensity, and magnitude of necessary exercise, are present; however, high-impact activities (e.g., running, plyometrics, and jumping) are generally considered more osteogenic than lower-impact activities (e.g., cycling and swimming). In impact exercises, such as running/HIIT, ground reaction forces constitute peak force exerted on the skeleton, whereas in low-impact exercises, such as MST, the peak force is generated through muscle work (20, 21). Although HIIT and MST subsequently differ in skeletal loading characteristics, both training forms can be regarded as being osteogenic (12, 17, 18, 22).

Circulating bone turnover markers (BTMs) reflect dynamic changes and provide a metabolic image of bone metabolism (23). The recommended BTMs in clinical practice are serum levels of resorption marker C-terminal telopeptide of type 1 collagen (CTX-I), a product of the enzymatic degradation of collagen fibers in bone, and total procollagen of type 1 N-terminal propeptide (P1NP), a sensitive marker for processes in proliferating osteoblast (23–25). Osteocalcin (OC), which is synthesized during bone formation by mature osteoblasts, is often regarded as a bone formation marker (26) but might also be released during bone resorption in both its intact and fragmented forms and may therefore indicate general bone remodeling (27).

Bone resorption and formation are orchestrated by many substances, and measurements of these may yield insight into the mechanisms for alterations in serum levels of BTMs. Sclerostin is an osteocyte-specific secreted potent inhibitor of bone formation *via* inhibition of the Wnt/β-catenin pathway. Immobilization is associated with a rise in serum sclerostin and decreased BMD (28).

Lipocalin-2 (LCN2) has been proposed to act as an osteoblast mechano-responding gene, upregulated in osteoblasts during microgravity and correlated with poor osteoblast activity (29). Serum LCN2 was increased after 14 days in a bed rest study with male subjects, and *Lcn2* expression was upregulated in bones in different mouse models of unloading, which could be counteracted by physical activity (30). In contrast, serum LCN2 was found to increase after high-intensity acute exercise in young and middle-aged male runners (31).

A systematic review of BTMs in subjects with osteoporosis revealed possible exercise benefits in terms of improving bone formation and decreasing bone resorption biomarkers (32). Whereas some studies have reported beneficial acute effects of exercise on BTMs (17, 22, 33), a direct comparison of heavy resistance training and aerobic running exercise has not yet been performed under strictly controlled conditions. Hack squat ST and treadmill HIIT are two well-studied training interventions that are highly relevant in the context of musculoskeletal and cardiometabolic health (11–16). Also, the acute response of these interventions on other mechano-responsive biomarkers, such as sclerostin and LCN2, has been less investigated, along with possible sex and age differences.

In the current study, we aimed to characterize the respective responses of hack squat ST and treadmill HIIT across age and gender. Specifically, we sought to investigate the acute exercise-induced effects of these two interventions on serum BTMs (CTX-1 and P1NP), OC, sclerostin, and LCN2 in men and women 21 to 30 years of age and elderly men 63 to 74 years of age.

## Methods

### Participants and Study Design

This was a three-group crossover within-subjects design study. Recruitment of participants took place from September 2018 to February 2019, through posters on local gyms and at the University of Science and Technology, Trondheim, Norway. The study was approved by the Regional Ethics Committee for Medical and Health Research (REK2018/926) and was performed in accordance with the Declaration of Helsinki.

A total of 53 men and women volunteered to participate in this study (19 young women (age 22–31 years), 20 young men (age 21–30 years), and 14 elderly men (age 63–74 years). Inclusion criteria were as follows: 1) did not suffer any significant illness relevant to the study, 2) free from chronic conditions and injuries that could influence blood samples or prevent the participants in performing the physical tests, and 3) not taking dietary supplements known to affect bone metabolism. All participants agreed to take part in the study by signing a consent form.

**Figure 1:**
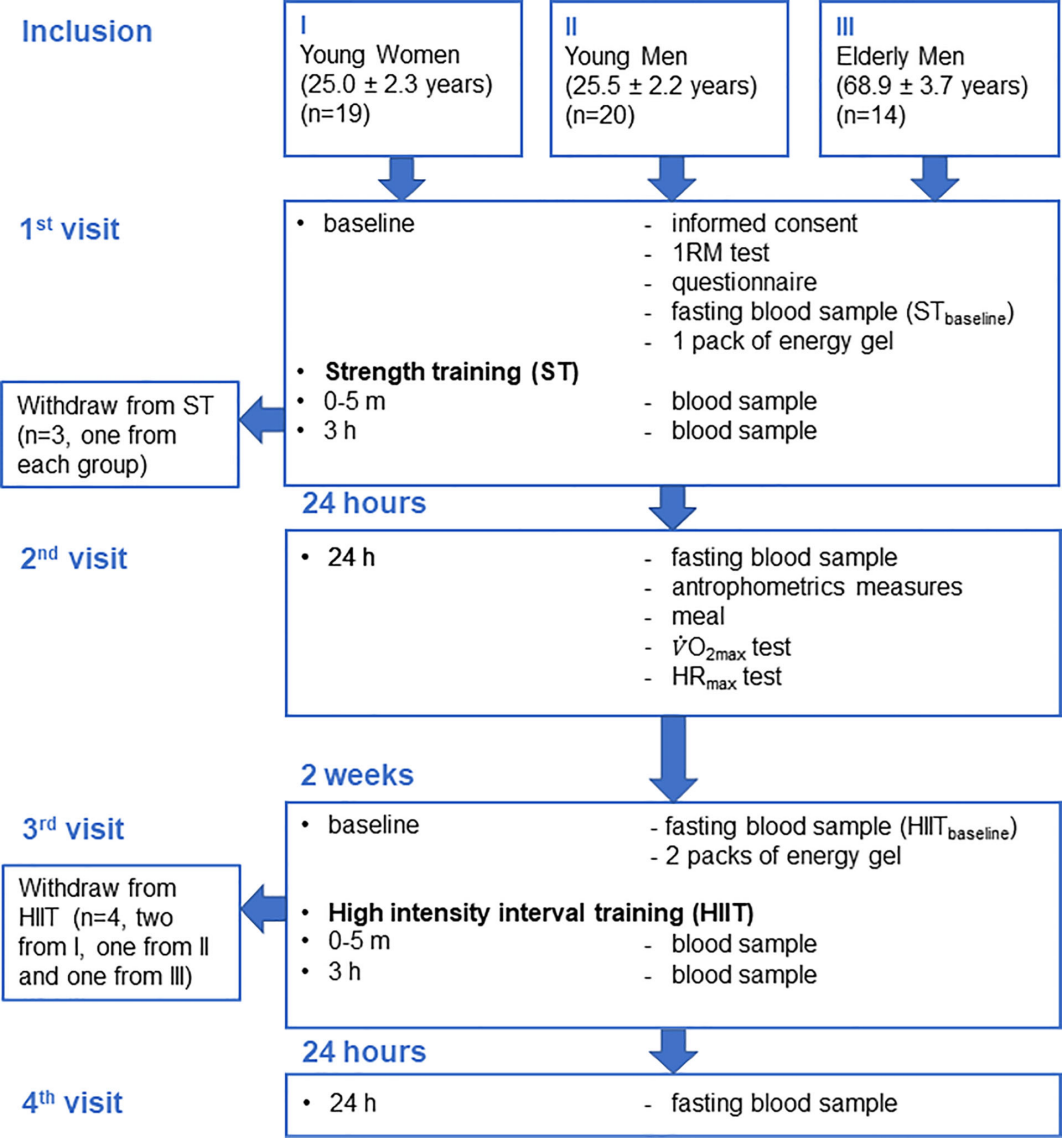
Flowchart of the study.

A flowchart of the study is presented in **Figure 1**. Each participant performed two training sessions, ST and HIIT, separated by 2 weeks. During both training sessions, a total of four blood samples were collected from each participant: at baseline (pre), immediately (0–5 min, post), and 3 and 24 h after training. The training sessions were performed in a supervised laboratory setting, and participants were asked to fast overnight, avoid dietary supplements, and exercise 48 h before the sessions. At the first visit (between 08:00 and 09:00), participants signed an informed consent form and answered a questionnaire regarding lifestyle, medical history, and use of oral contraceptives (young women). Overnight fasting baseline (ST_baseline_) blood samples were drawn, and participants were tested for 1 repetition maximum (1RM) before the ST session, followed by post and 3 h after ST blood sampling. Between 08:00 and 10:00 the following day (second visit), blood samples were collected 24 h after ST, following an overnight fast. Body weight was obtained using an electronic scale, height was measured using a wall-mounted stadiometer, and body mass index (BMI; kg/m^2^) was calculated. Further, after participants had a meal, their maximal oxygen uptake (V̇O_2max_) and maximal heart rate (HR) (HR_max_) were measured. Two weeks later, between 08:00 and 09:00 (third visit), overnight fasting baseline (HIIT_baseline_) blood samples were drawn, and participants took part in the HIIT session, followed by post and 3 h after HIIT blood sampling. Between 08:00 and 10:00 the following day (fourth visit), the blood samples after overnight fasting 24 h after HIIT were collected.

### One Repetition Maximum

1RM was tested in a seated leg press apparatus (TechnoGym, Cesena, Italy), according to a protocol by the American College of Sports Medicine (ACSM) (34). The protocol consisted of two warm-up sets and a maximum of five attempts to determine the maximum load of a single dynamic leg press movement. Participants were instructed to perform a leg press with a knee joint angle of 90°. The first warm-up set consisted of 5–10 repetitions at 40%–60% of assumed 1RM. The second warm-up set consisted of 3–5 repetitions at 60%–80% of assumed 1RM. After the second warm-up, the load was increased to the expected 1RM and the first attempt started. If the participant was able to complete one repetition with the given load, a new attempt with increased load was initiated after 3–5 min of rest, which was repeated until failure.

### Maximal Oxygen Uptake and Maximal Heart Rate

Maximal oxygen uptake (V̇O_2max_) was measured on a treadmill (Woodway PPS Med, Weil am Rhein, Germany) with a Metamax II Portable device (Cortex, Leipzig, Germany). Participants warmed up for 10 min before the test. During the test, the minimum inclination on the treadmill was 5.3%, and the speed was increased every 2–3 min until the participant reached exhaustion. V̇O_2max_ was accepted when it leveled off despite further increases in workload and verified by lactate levels ≥8.0 mmol/L and respiratory exchange ratio ≥1.05. Lactate was measured with a biochemical analyzer (EKF Biosen C-line Diagnostics, Barleben, Germany), and HR was assessed with a Polar M200 monitor (Kempele, Finland). Maximal HR (HR_max_) was set as the highest HR registered + 3 beats per minute.

### Blood Sampling and Biochemical Analyses

Blood samples were collected by standard venipuncture. Blood was collected in vacuum tubes, let sit for 30 min at room temperature, and centrifuged (3,000 *g*/4°C/10 min) before serum was aliquoted and stored at −80°C until further analyses. Serum albumin, creatinine, and total calcium (Ca) were analyzed with accredited analyses at the Department of Biochemistry, St. Olavs University Hospital, Trondheim, Norway. The bone turnover markers (BTMs) in serum resorption marker C-terminal telopeptide of type 1 collagen (CTX-I) and formation marker total procollagen of type 1 N-terminal propeptide (P1NP) were measured by accredited electrochemiluminescence immunoassays (Roche Cat# 11972308122, RRID:AB_2905599 and Roche Cat# 03141071190, RRID:AB_2782967, respectively), and total osteocalcin (OC) (intact (1-49) and large fragments (1-43)) by an accredited chemiluminescence assay (DiaSorin, Cat# 310950, RRID:AB_2917975;LIAISON® OSTEOCALCIN 1-49) at the Hormone Laboratory, Oslo University Hospital, Oslo, Norway. Serum sclerostin was analyzed by a human sclerostin ELISA kit (Biomedica Cat# BI-20492, RRID : AB_2894889), and serum LCN2 was analyzed by a human LCN2/NGAL DuoSet ELISA kit (R&D Systems Cat# DLCN20, RRID : AB_2894833). Analyses were done according to the manufacturers’ instructions.

### Strength Training

At the first visit, after the overnight fasting baseline_ST_ blood samples were drawn, participants were offered one pack of energy gel, containing pure carbohydrates (60 ml, 87 kcal) (Go Isotonic Energy, Science in Sport, London, UK) before the ST session. The training was conducted in the same leg press apparatus as used for the 1RM testing procedure, consisting of 4 sets of 8–10 repetitions until failure, with 2–3-min rest between sets. The load was 80% of the participants measured 1RM. The training session was performed with a slow eccentric movement down to a 90° knee angle and maximal intended velocity in the concentric movement. Participants’ mean values of 1RM are presented in **Table 1**.

**Table 1:** Participants’ baseline characteristics and measures.

	I, Young women (n = 19)		II, Young men (n = 20)		III, Elderly men (n = 14)	
	Mean ± SD	Range	Mean ± SD	Range	Mean ± SD	Range
**Age (years)**	25.0 ± 2.3	22–30	25.5 ± 2.2	21–30	68.9 ± 3.7	63–74
**Weight (kg)**	65.6 ± 7.5	46.6–75.5	77.2 ± 10.3	62.8–103	79.2 ± 10.4	61.2–98.5
**Height (cm)**	169 ± 5.4	158–181	181 ± 6.2	168–193	180 ± 6.7	169–193
**BMI (kg·m^−2^)**	23.0 ± 2.9	16–28	23.5 ± 2.5	19–28	24.4 ± 2.5	20–28
**V̇O_2max_**						
**(L·min^−1^)**	3.3 ± 0.5	2.26–4.05	4.8 ± 0.6	3.76–5.85	3.5 ± 0.5	2.64–4.81
**(mL·kg^−1^·min^−1^)**	50.5 ± 6.3	35.7–60.3	62.6 ± 8.3	46.9–77.0	42.5 ± 5.4	34.0–51.3
HRmax (bmp)*	195 ± 9	179–210	196 ± 7	182–207	169 ± 17	143–197
1RM (kg)**	104 ± 25	64–140	134 ± 28	80–194	130 ± 17	98–164
**sAlbumin (g/L)**	44 ± 1.8	41–47	46 ± 1.8	42–49	43 ± 1.8	40–46
Reference***		36–48		36–48		36–45
**sCreatinine (µmol/L)**	73.8 ± 12.5	58–105	78.8 ± 7.8	63–90	84.3 ± 13.4	61–106
Reference***		45–90		60–105		60–105
**sCalcium (nmol/L)**	2.35 ± 0.04	2.26–2.46	2.40 ± 0.08	2.14–2.50	2.36 ± 0.06	2.22–2.44
Reference***		2.15–2.51		2.15–2.51		2.15–2.51

Data are in mean ± SD and range (minimum to maximum). Baseline serum levels are calculated mean values from ST_baseline_ and HIIT_baseline_.

BMI, body mass index; V̇O_2max_, maximal oxygen uptake; HR_max_, maximal heart rate in beats per minute; 1RM, one repetition maximum; s, serum.

*Data of one young woman are missing.

**Data of one young woman and one young man are missing.

***Clinical reference ranges for validated analyses from Department of Biochemistry, St. Olavs University Hospital, Trondheim, Norway.

### High-Intensity Interval Training

At the third visit, 2 weeks after the ST session, the participants took part in a HIIT session on a treadmill, as previously described (35). The participants were offered two packs of energy gel before this session. Participants warmed up for 10 min before performing 4 × 4 min intervals, with 3 min of active rest, on a treadmill with a 5.3% incline. During the intervals, the treadmill speed was set to a target intensity of 90%–95% of each participant’s HR_max_, and during active rest periods, an intensity was set corresponding to 70% of HR_max_. Participants’ mean values of HR_max_ are presented in **Table 1**.

### Corrections for Plasma Volume Changes

Plasma volume changes (ΔPV) can occur during and after exercise due to transient fluid shifts (hemodilution and hemoconcentration) (36). The ΔPV can affect the interpretation of biochemical measurements in blood, and changes in the concentrations of biomarkers in blood should therefore be adjusted for ΔPV in exercise studies (36, 37). The standard method for calculation of %ΔPV has been the Dill and Costill equation (37), requiring hematocrit (Htc) and hemoglobin (Hb) measurements. Changes in serum total calcium levels after exercise have been found to correlate well with %ΔPV and can therefore be used as a hemoconcentration biomarker in exercise studies (38). In the current study, all serum analyses at post-test, 3 h, and 24 h were corrected for %ΔPV using %Δ in [Ca] from corresponding baseline values, before comparisons. The following equations for corrections were used:


$$\text{A})\%\text{Δ}PV_{t1}=100*\left(\frac{{\left[Ca\right]}_{t1}-{\left[Ca\right]}_{baseline}}{{\left[Ca\right]}_{baseline}}\right)$$

$$\text{B})\;\left[bone\;marker\;\right]corrected_{t1}={\left[bone\;marker\;\right]}_{t1}*\left(1-\left(\frac{\%\text{Δ}PV_{t1}}{100}\right)\right)$$

where *t*_1_ represents the time point for measurements (post-test, 3 h, or 24 h) after baseline in the applicable training mode. All serum analyses at post-tests and 3 and 24 h after the training intervention were performed with serum levels corrected for ΔPV as described (denoted as bone marker_c_).

### Statistical Analyses

Three missing values at individual time points (due to participant’s absence) and one extreme outlier were replaced with the median value of the corresponding time point for analyses.

Data were assessed for normality using a Shapiro–Wilk test and checked for outliers by visual inspection by Q-Q plots. Skewness and kurtosis were analyzed by g1 and z-score.

A one-way ANOVA with a *post*-*hoc* Tukey’s multiple-comparison test with computed multiplicity adjusted p-values for each comparison was used to examine group differences in mean baseline values between the two young groups and the two male groups (calculated as mean values from ST_baseline_ and HIIT_baseline_) of BTMs, OC, sclerostin, and LCN2.

Two-way repeated-measures ANOVA (two-way-RM-ANOVA) with Geisser–Greenhouse correction to adjust for lack of sphericity was used to examine time and group main effects for CTX-1_c_, P1NP_c_, P1NP_c_/CTX_c_ ratio, OC_c_, sclerostin_c,_ and LCN2_c_ in each training mode. In case of a significant simple main effect of time, a within-group *post*-*hoc* Tukey’s multiple-comparison test with computed multiplicity adjusted p-values for each comparison was performed. Thereafter, a two-way-RM-ANOVA with Geisser–Greenhouse correction to adjust for lack of sphericity was used to examine the time and training mode main effects for each serum marker within each participant group. In case of significant training mode main effects, a Šidák multiple-comparison *post*-*hoc* test, with computed multiplicity adjusted p-values for each comparison, was performed for comparison of training mode at each time point.

For serum markers that exhibited a significant simple main effect of time, Pearson’s correlation coefficients were used to determine the relationship between percentage change in plasma volume-corrected serum marker concentrations from baseline and the participants’ 1RM and V̇O_2max_.

All statistical analyses were performed using SPSS (IBM SPSS, Inc., version 27, 2020) and GraphPad Prism (GraphPad Software, Inc., version 9.2.0, 2021). Figures were made in GraphPad Prism.

## Results

### Participants and Baseline Measurements

A flowchart of the study design and participants is shown in **Figure 1**. Values for serum BTMs and related markers were normally distributed, with the expectation of one extreme outlier value for measurements of LCN2_c_, which were replaced with the median value of the corresponding time point for further analyses. The participants’ baseline characteristics, V̇O_2max_, HR_max_, and 1RM are presented in **Table 1**. All three groups of participants were healthy, meaning an average BMI within the accepted normal range and fasting mean baseline serum levels of albumin, creatinine, and calcium within the clinical reference ranges (**Table 1**). A total of 396 blood samples (n = 50 and 4 time points in ST and n = 49 and 4 time points in HIIT) were included in the analyses.

Serum values for each group and exercise mode of calcium [Ca] and the %Δ[Ca] from the baseline used for correction of ΔPV (%ΔPV) are shown in **Table 2**.

**Table 2:** Participants’ serum total calcium [Ca] and %Δ[Ca] from baseline for correction of plasma volume changes.

Calcium (nmol/L)	I, Young women		II, Young men		III, Elderly men	
	ST (n = 18)	HIIT (n = 17)	ST (n = 19)	HIIT (n = 19)	ST (n = 13)	HIIT (n = 13)
**Baseline (BL)**	2.37 ± 0.07	2.34 ± 0.05	2.39 ± 0.08	2.42 ± 0.08	2.36 ± 0.05	2.35 ± 0.08
**Post test**	2.43 ± 0.09	2.49 ± 0.06	2.52 ± 0.08	2.47 ± 0.08	2.44 ± 0.05	2.40 ± 0.07
**%Δ[Ca] from BL**	6.62 ± 2.89	2.69 ± 2.40	5.25 ± 1.95	2.28 ± 1.97	3.61 ± 1.92	1.68 ± 1.93
**3 h**	2.40 ± 0.06	2.42 ± 0.05	2.45 ± 0.08	2.47 ± 0.09	2.38 ± 0.06	2.39 ± 0.08
**%Δ[Ca] from BL**	3.59 ± 2.90	1.71 ± 1.96	2.26 ± ´3.19	2.07 ± 2.7	0.92 ± 1.68	0.99 ± 1.90
**24 h**	2.38 ± 0.06	2.39 ± 0.07	2.41 ± 0.07	2.41 ± 0.07	2.34 ± 0.07	2.32 ± 0.06
**%Δ[Ca] from BL**	2.10 ± 2.60	0.47 ± 2.05	0.52 ± 3.10	−0.52 ± 3.09	−0.95 ± 1.72	−1.37 ± 2.00

Data are in mean ± SD.

ST, strength training; HIIT, high-intensity interval training; BL, baseline.

**Figure 2:**
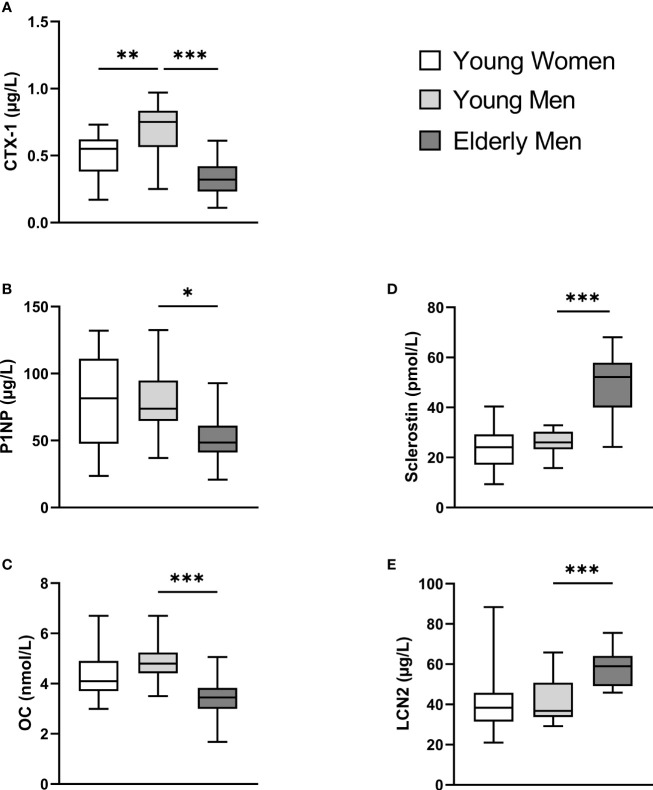
Participants’ baseline serum levels (calculated mean values from ST_baseline_ and HIIT_baseline_) of C-terminal telopeptide of type 1 collagen (CTX-I) **(A)**, total procollagen of type 1 N-terminal propeptide (P1NP) **(B)**, total osteocalcin (OC) **(C)**, sclerostin **(D)**, and lipocalin-2 (LCN2) **(E)**. A one-way ANOVA with a *post*-*hoc* Tukey’s multiple-comparison test with computed multiplicity adjusted p-values for comparisons was used to examine group differences between mean baseline values between young women versus young men and young versus elderly men. Data are presented as boxplot with range (minimum to maximum). *p < 0.05, **p < 0.01, ***p < 0.001.

The mean baseline levels of BTMs, OC, sclerostin, and LCN2 are presented in **Figure 2**. Young men had higher baseline CTX-1 levels than young women (mean difference (MD) = 0.17 µg/L, **Figure 2A**). Elderly men had lower baseline BTMs (CTX-1 and P1NP) and OC levels as compared to the young male group (CTX-1, MD = 0.35 µg/L; P1NP, MD = 28.6 µg/L; OC, MD = 1.4 nmol/L, **Figures 2A–C**). Elderly men had higher baseline sclerostin and LCN2 levels as compared to young men (sclerostin, MD = 23.2 pmol/L; LCN2, MD = 17.4 µm/L, **Figures 2D, E**).

### Sex/Age and Time Effects

There were significant main effects of group (sex/age)-by-time on plasma volume-corrected levels of CTX-1_c_, P1NP_c_/CTX-1_c_ ratio, and sclerostin_c_ in ST (F(6,141) = 5.48, 3.61, and 2.18, respectively, p < 0.05 for all) and for CTX-1_c_ and P1NP_c_ in HIIT (F(6,138) = 7.81, p = 0.0001, and F(6,138) = 4.35, p = 0.0005, respectively). There was a significant simple main effect of group on all analyzed plasma volume-corrected serum markers (F(2,47) ranging from 3.40 to 39.9, p < 0.05 for all in ST and F(2, 44) ranging from 3.43 to 41.64, p < 0.05 for all in HIIT).

There was a significant simple main effect of time on most of the plasma volume-corrected serum markers in both exercise modes, and *post*-*hoc* Tukey multiple-comparison tests of the simple effect of time (from baseline) within-group and exercise mode showed the following.

#### Group I, Young Women

**Figure 3:**
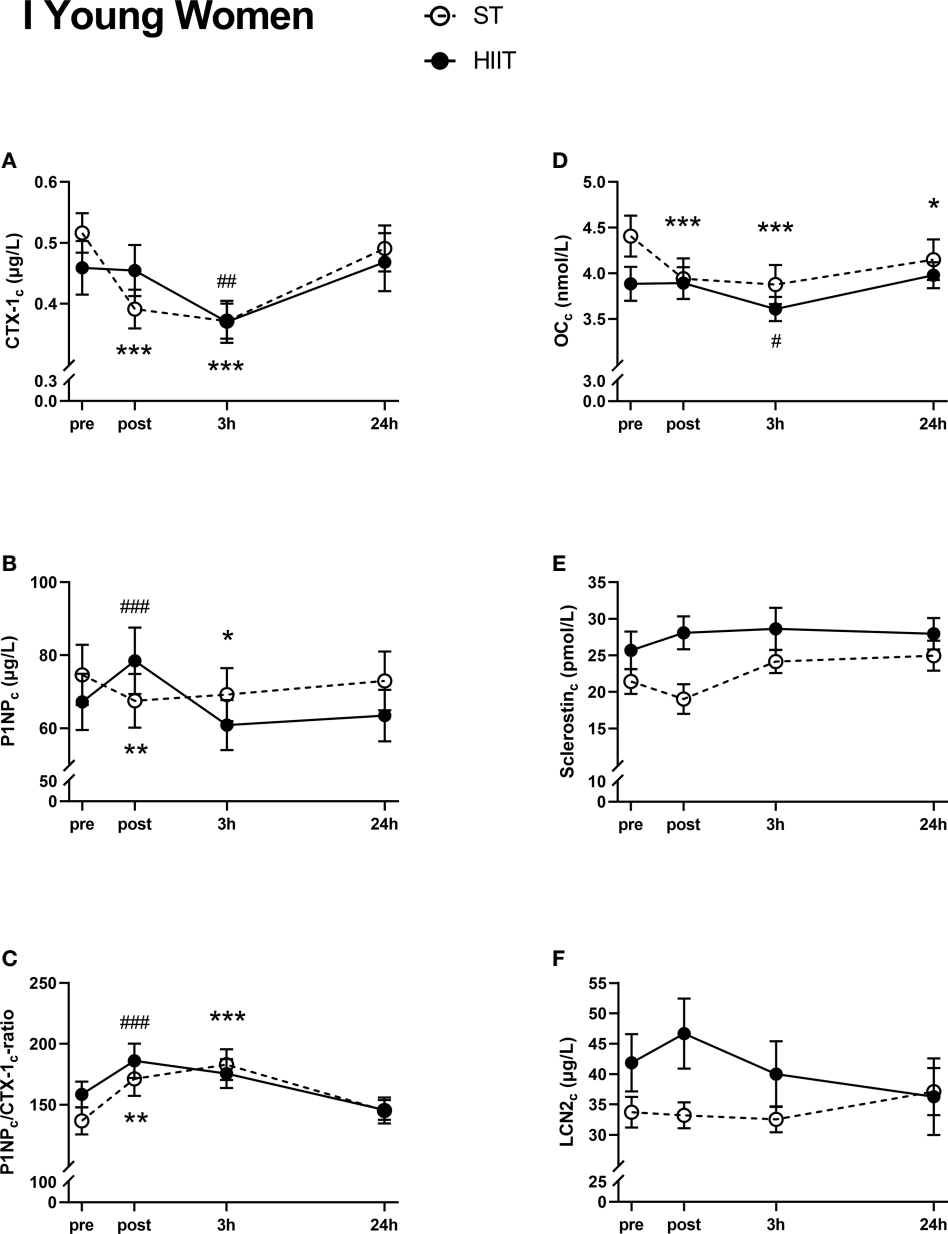
Plasma volume-corrected serum levels of C-terminal telopeptide of type 1 collagen (CTX-I_c_) **(A)**, total procollagen of type 1 N-terminal propeptide (P1NP_c_) **(B)**, PINP_c_/CTX-1_c_ **(C)**, total osteocalcin (OC_c_) **(D)**, sclerostin_c_ **(E)**, and lipocalin-2 (LCN2_c_) **(F)** in young women at baseline (pre), immediately after the strength training (ST) or high-intensity interval training (HIIT) sessions (post), and 3 and 24 h after exercise. Data are in mean ± SEM. *p < 0.05, **p < 0.01, ***p < 0.001 versus baseline after ST and ^##^p < 0.01, ^###^p < 0.001 versus baseline after HIIT (two-way repeated-measures ANOVA and a within-group *post*-*hoc* Tukey’s multiple-comparison test with computed multiplicity adjusted p-values for each comparison).

Serum CTX-1_c_ decreased from baseline to post-test after ST training and after 3 h after both training modes, before returning to baseline levels after 24 h (**Figure 3A**). Serum P1NP_c_ decreased from baseline to post-test and 3 h after ST training, increased from baseline to post-test after HIIT, and returned to baseline levels after 24 h for both exercise modes (**Figure 3B**). Serum P1NP_c_/CTX-1_c_ ratio increased from ST baseline to post-test after 3 h and from HIIT baseline to post-test and returned to baseline levels after 24 h for both exercise modes (**Figure 3C**). Serum OC_c_ decreased from baseline ST to post-test after 3 and 24 h. After HIIT, serum OC_c_ decreased from baseline to 3 h but returned to baseline values after 24 h (**Figure 3D**). There were no significant time effects on the serum levels of neither sclerostin_c_ nor LCN2_c_ after either exercise mode in the young female group (**Figures 3E, F**).

#### Group II, Young Men

**Figure 4:**
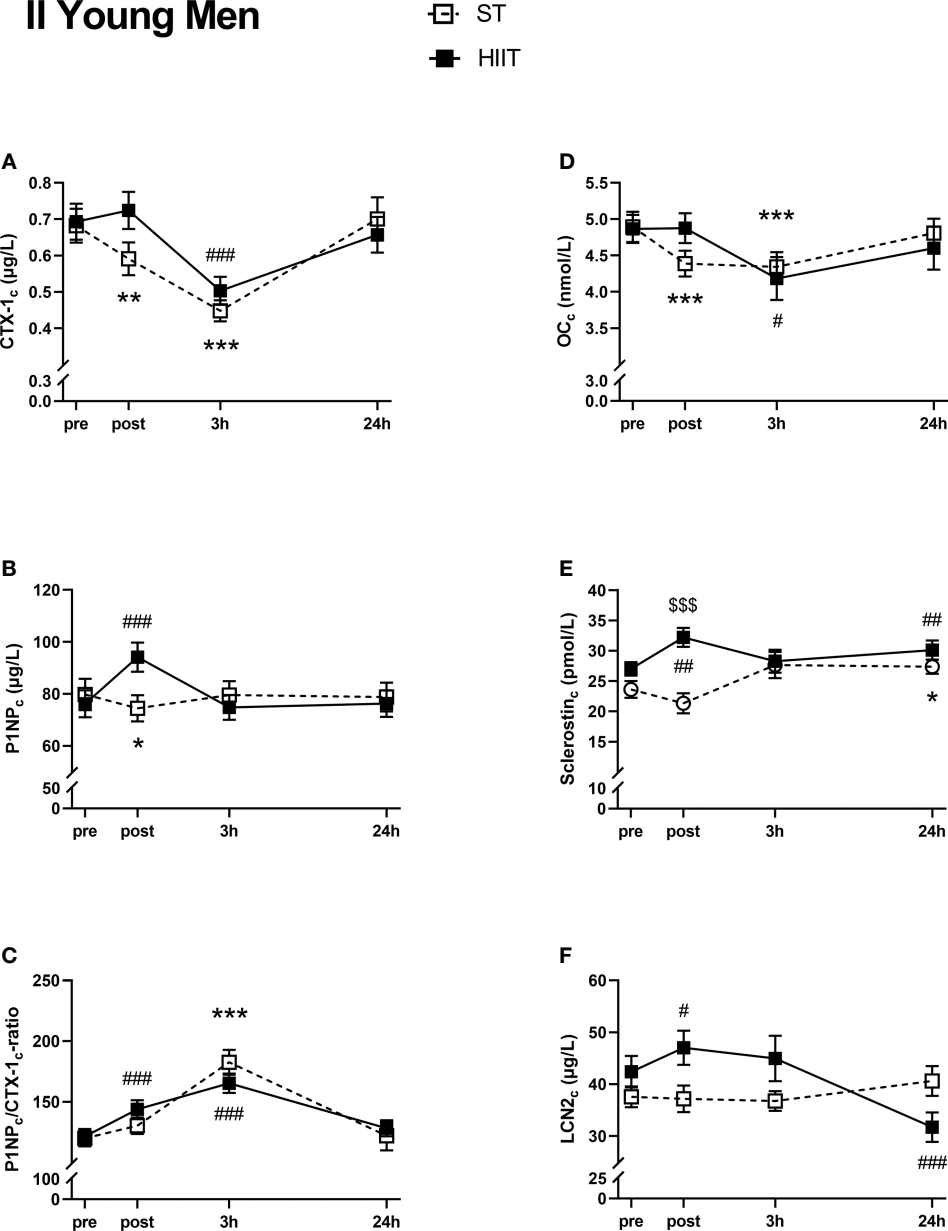
Plasma volume-corrected serum levels of C-terminal telopeptide of type 1 collagen (CTX-I_c_) **(A)**, total procollagen of type 1 N-terminal propeptide (P1NP_c_) **(B)**, PINP_c_/CTX-1_c_ **(C)**, total osteocalcin (OC_c_) **(D)**, sclerostin_c_ **(E)**, and lipocalin-2 (LCN2_c_) **(F)** in young men at baseline (pre), immediately after the strength training (ST) or high-intensity interval training (HIIT) sessions (post), and 3 and 24 h after exercise. Data are in mean ± SEM. *p < 0.05, **p < 0.01, ***p < 0.001 versus baseline after ST and ^#^p < 0.05, ^##^p < 0.01, ^###^p < 0.001 versus baseline after HIIT (two-way repeated-measures ANOVA and a within-group *post*-*hoc* Tukey’s multiple-comparison test with computed multiplicity adjusted p-values for each comparison). ^$$$^p < 0.001 ST versus HIIT training modes (two-way repeated-measures ANOVA and a Šidák multiple-comparison *post*-*hoc* test for comparison of the two training modes at each time point).

There was a significant decrease in serum CTX-1_c_ from ST baseline to post-test and 3 h and from HIIT baseline to 3 h (**Figure 4A**). Serum CTX-1_c_ returned to baseline values after 24 h for both exercise modes. Serum P1NP_c_ decreased from ST baseline to post-test and increased from HIIT to post-test (**Figure 4B**). After 24 h, P1NP_c_ returned to baseline levels. The serum P1NP_c_/CTX-1_c_ ratio was increased from baseline after 3 h in ST and from HIIT baseline to post-test and after 3 h and returned to baseline values for both after 24 h (**Figure 4C**). Serum OC_c_ decreased from ST baseline to post-test and after 3 h and from HIIT baseline to 3 h before returning to baseline values after 24 h (**Figure 4D**). Serum sclerostin_c_ increased from ST baseline to 24 h and increased from HIIT baseline to post-test and after 24 h (**Figure 4E**). There were no significant time effects on serum LCN2_c_ after ST, but LCN2_c_ increased from HIIT baseline to post-test and was thereafter decreased compared to baseline values after 24 h (**Figure 4F**).

#### Group III, Elderly Men

**Figure 5:**
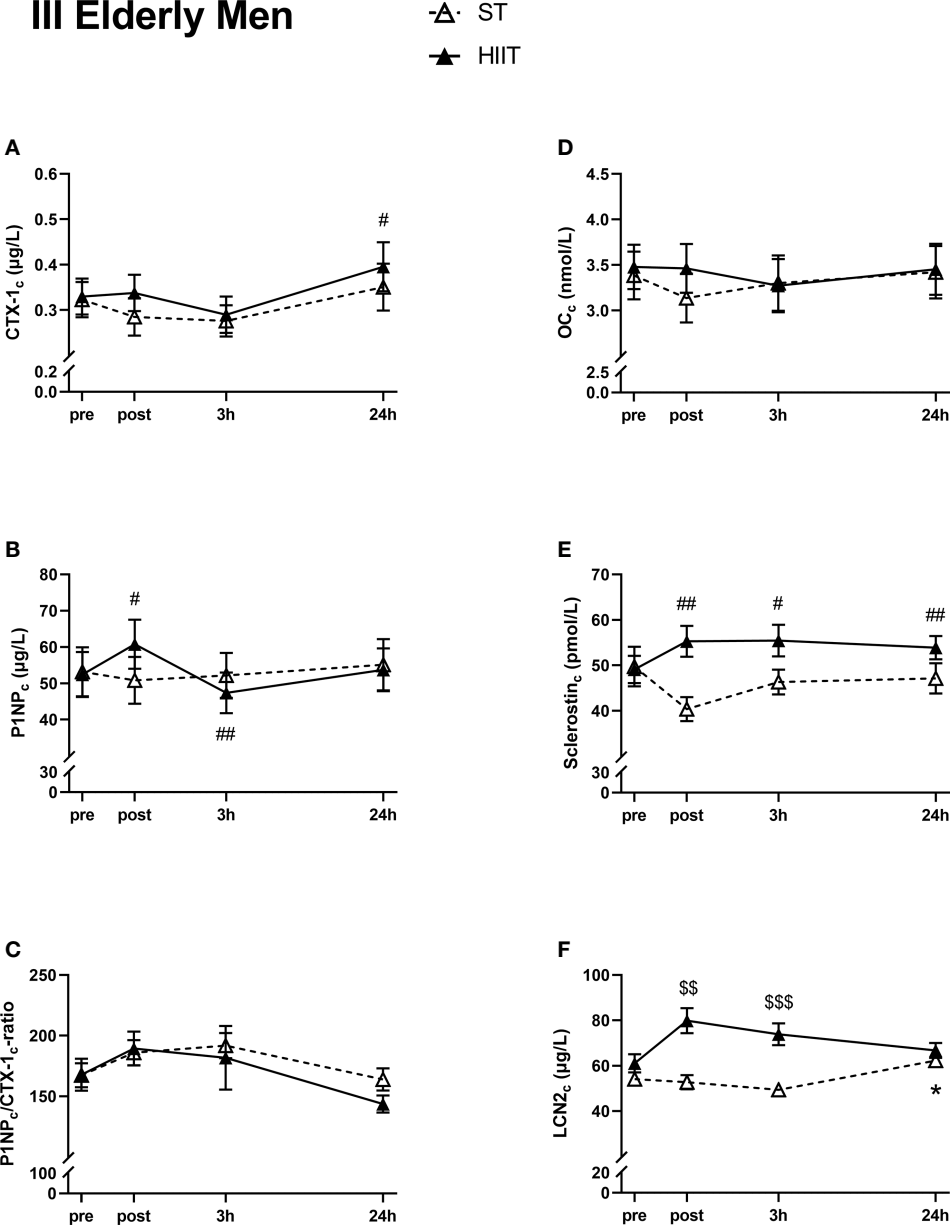
Plasma volume-corrected serum levels of C-terminal telopeptide of type 1 collagen (CTX-I_c_) **(A)**, total procollagen of type 1 N-terminal propeptide (P1NP_c_) **(B)**, PINP_c_/CTX-1_c_ **(C)**, total osteocalcin (OC_c_) **(D)**, sclerostin_c_ **(E)**, and lipocalin-2 (LCN2_c_) **(F)** in elderly men at baseline (pre), immediately after the strength training (ST) or high-intensity interval training (HIIT) sessions (post), and 3 and 24 h after exercise. Data are in mean ± SEM. *p < 0.05 versus baseline after ST and ^#^p < 0.05, ^##^p < 0.01 versus baseline after HIIT (two-way repeated-measures ANOVA and a within-group *post*-*hoc* Tukey’s multiple-comparison test with computed multiplicity adjusted p-values for each comparison). ^$$^p < 0.01, ^$$$^p < 0.001 ST versus HIIT training modes (two-way repeated-measures ANOVA and a Šidák multiple-comparison *post*-*hoc* test for comparison of the two training modes at each time point).

There were no significant time effects on serum CTX-1_c_, except for a significant increase from baseline to 24 h after HIIT (**Figure 5A**). No time effects were seen in serum P1NP_c_ levels after ST, while there was a significant increase from HIIT baseline to post-test followed by a decrease after 3 h, before returning to baseline values after 24 h (**Figure 5B**). There were no significant time effects on serum P1NP_c_/CTX-1_c_ ratio or serum OC_c_ in neither of the two exercise modes (**Figures 5C, D**). Serum sclerostin_c_ was not affected after ST at any measured time points, unlike after HIIT, where sclerostin_c_ was significantly increased at post-test, after 3 and 24 h compared to baseline (**Figure 5E**). Serum LCN2_c_ was not significantly affected by time after exercise, except for a small increase after 24 h compared to ST baseline (**Figure 5F**).

### Exercise Mode and Time Effects

Since there was a significant main effect of the group on all plasma volume-corrected serum markers, the effect of exercise mode by time was analyzed separately in the groups I, II, and III for each serum analysis.

#### Group I, Young Women:

The two-way RM ANOVA showed no significant main effects of the exercise modes ST and HIIT for any of the plasma volume-corrected measured markers (CTX-1_c_, F(1,33) = 0.008, p = 0.93; P1NP_c_, F(1,33) = 0.106, p = 0.75; P1NP_c_/CTX-1_c_ ratio, F(1,33) = 0.224, p = 0.64; OC_c_, F(1,33) = 0.842, p = 0.37; sclerostin, F(1,33) = 3.52, p = 0.07; LCN2_c_, F(1,33) = 1.94, p = 0.17).

#### Group II, Young Men

There were no significant main effects of the exercise modes for any of the plasma volume-corrected serum markers, except for sclerostin_c_ (CTX-1_c_, F(1,36) = 0.407, p = 0.53; P1NP_c_, F(1,36) = 0.085, p = 0.77; P1NP_c_/CTX-1_c_ ratio, F(1,36) = 0.009, p = 0.92; OC_c_, F(1,36) = 0.008, p = 0.93; sclerostin_c_, F(1,36) = 5.837, p = 0.021; LCN2_c_, F(1,36) = 0.874, p = 0.36).

A Šidák multiple-comparison *post*-*hoc* test for comparison of exercise mode at each time point showed significantly higher sclerostin_c_ in HIIT compared to ST at post-test immediately after exercise (MD 10.85 pmol/L) (**Figure 4E**).

#### Group III, Elderly Men

There was a significant main effect of exercise mode for LCN2_c_, but not for CTX-1_c_, P1NP_c_, OC_c_, and sclerostin_c_ (CTX-1_c_, F(1,24) = 0.261, p = 0.61; P1NP_c_, F(1,24) = 0.007, p = 0.93; P1NP_c_/CTX-1_c_ ratio, F(1,24) = 0.295, p = 0.66; OC_c_, F(1,24) = 0.081, p = 0.78; sclerostin_c_, F(1,24) = 3.243, p = 0.08; LCN2_c_, F(1,24) = 3.567, p < 0.001).

A Šidák multiple-comparison *post*-*hoc* test for comparison of exercise mode at each time point showed significantly higher levels of LCN2_c_ in HIIT versus ST at post-test immediately after exercise (MD 27.21 µg/L), as well as 3 h after exercise (MD 24.56 µg/L) (**Figure 5F**).

### Correlations

For plasma volume-corrected serum markers that exhibited a significant simple main effect of time, we analyzed the relationship between the percentage change and 1RM and V̇O_2max_. For young women, we found a negative correlation between 1RM and CTX-1_c_ percentage changes from baseline to post-test and 3 h after ST (r = −0.571, p = 0.013, and r = −0.471, p = 0.049, respectively) and a positive correlation between 1RM and the percentage changes in P1NP_c_ from baseline to post-test after HIIT (r = 0.645, p = 0.013). The change in OC_c_ was negatively correlated to 1RM at post-test after ST (r = −0.664, p = 0.003) and positively correlated to 1RM at post-test and 3 h after HIIT (r = 0.596, p = 0.025 and r = 0.641, p = 0.014, respectively), while changes in sclerostin_c_ at post-test after HIIT were positively correlated to 1RM (r = 0.645, p = 0.013) in the young female group.

The plasma volume-corrected percentage changes in serum markers from baseline to post-test and 3 and 24 h after ST and HIIT, were not correlated to V̇O_2max_ in neither of the three groups nor with 1RM in the young and elderly male groups.

## Discussion

In the current study, we investigated the acute effects of two well-recognized training interventions for cardiometabolic and musculoskeletal health, namely, treadmill HIIT and hack squat ST. The respective responses to bone turnover and mechano-responsive biomarkers in young men and women, and in elderly men, were explored.

The elderly men displayed significantly lower baseline values of the BTMs CTX-1 and P1NP, as well as OC, whereas LCN2 and sclerostin levels were higher, the latter by twofold, compared to those in the young male participants. Both training modes induced an acute decrease in plasma volume-corrected CTX-1_c_ in the young groups, whereas PINP_c_ levels were divergently affected, with HIIT promoting a transient increase and ST a transient decrease. Both training modes evoked a rise in the ratio of P1NP_c_/CTX-1_c_ at post-test and/or after 3 h in the young groups, but not in elderly men. A significant increase in sclerostin_c_ was seen in young and elderly men after HIIT, which lasted for 24 h. Moreover, LCN2_c_ levels were elevated immediately after HIIT in young men but decreased after 24 h as compared to baseline. Correlations between simple time effects changes in BTMs and related bone markers and the participants’ muscle strength and cardiovascular fitness were solely seen in the young female group.

### Age and Sex Differences in Baseline Levels of Bone Turnover Markers, Osteocalcin, Sclerostin, and Lipocalin-2

In line with previous studies, we observed significantly lower baseline levels of BTMs and OC in elderly men compared to young men, reflecting an age-induced decline in bone turnover (39). This could partly be due to the high level of baseline sclerostin in this group, as sclerostin acts as a strong inhibitor of bone formation. The role of LCN2 in bone is not fully settled, but it has been shown to inhibit osteoclast generation (40) and is associated with reduced osteoblast activity (29), which could reduce bone turnover. Baseline levels of sclerostin and LCN2 were higher in the elderly men compared to the young male participants. Moreover, young men displayed higher levels of CTX-1 than young women, confirming previous studies (41, 42), while baseline levels of P1NP, OC, sclerostin, and LCN2 did not differ between the young groups.

### Age and Sex Differences in Training-Induced Changes in Serum Markers

The alterations in BTMs after training were most pronounced in the young participants. This indicates that the acute effects of training on bone turnover decrease by age, as also suggested in a recent systematic review by Smith et al. (19). In both groups of young participants, we observed an immediate decrease in the serum bone resorption marker CTX-1_c_ after both training modes, which returned to baseline level after 24 h. A decline in P1NP_c_ levels was also seen in these groups after ST. Notably, P1NP_c_ levels increased transiently in all groups after HIIT. This was paralleled by a rise in sclerostin in the two male groups and by an increase in LCN2_c_ in the young male group. When calculating the ratio of P1NP_c_/CTX-1_c_, we observed an increase in both sexes, after both training modes, at post-test and/or after 3 h, suggesting a favorable acute effect of training on bone formation. In line with this, Whipple et al. found that resistance training with moderate intensity acutely reduced the serum bone resorption marker type I collagen N-telopeptide (NTX) for at least 8 h post-exercise in untrained young men (43). In contrast, Mieszkowski et al. reported that CTX-1 levels increased acutely after exercise in physically active young men, with no change in P1NP levels (44).

In contrast to the young participants, we observed no alterations after ST in elderly men, whereas HIIT induced altered CTX-1_c_ and P1NP_c_ levels. Moreover, no significant time effects were seen on P1NP_c_/CTX-1_c_ ratio after either exercise mode. These results are partly in line with a study by Maïmoun et al., who found no significant change in CTX-1 in elderly participants after a maximal incremental exercise test (45); however, both elderly men and women were included, and P1NP was not measured in this study. In continuation of this, a more recent study reported beneficial effects of plyometric exercise on the P1NP/CTX-1 ratio in premenopausal women, an effect that was blunted in postmenopausal women (33). The latter study reported the effect of BTMs as a formation/resorption ratio, using P1NP and CTX-1 (33). In the current study, we present P1NP/CTX-1 ratio, as part of a strategy to overcome the existing discrepancy between studies, as also suggested and used in several other reports (46–48).

We observed an acute decrease in OC_c_ at post-test and/or 3 h after both exercise modes in young participants, but not in the elderly men. Other studies show conflicting results regarding the effect of exercise on serum OC levels, as reviewed by Mohammad Rahimi et al. (49), making comparisons difficult. The interpretation of serum OC levels is further complicated by the fact that it exists in the circulation in two forms, namely, a carboxylated (cOC) form reflecting bone mineralization and an undercarboxylated (ucOC) form acting as a hormone, i.e., energy metabolism (50).

### Few Differences in Exercise Mode Effects on Serum Markers

The hack squat ST and treadmill HIIT induced similar effects on BTMs in young women and men. Potential differences between these exercise modes in the mechanisms accounting for the BTM response are still unclear. Treadmill HIIT yields a substantially higher metabolic load than hack squat ST. In the context of bone strain rate, however, running exercise (as in HIIT) is commonly regarded as an impact exercise, where ground reaction forces constitute the peak forces exerted on the skeleton, whereas ST provides peak forces through muscle work, being referred to as low-impact exercise (20, 21). A substantial amount of research has demonstrated that distinct changes in BTMs can occur following both high- and low-impact exercise, despite differences in metabolic load (20–22, 51). The same has also been reported with BMD adaptations from long-term training interventions (18). Previous studies of acute exercise have reported similar BTM responses as the results of our study, following resistance, plyometric, and endurance exercise, when investigated separately (17, 22, 51). Collectively, these findings suggest that BTMs can be acutely secreted from bone cells, reflecting changes in bone homeostasis induced by both high- and low-impact exercise despite differences in the initiating stimulus.

The BTM response was essentially similar after ST and HIIT among the young participants, whereas the immediate response in serum sclerostin_c_ differed, with an increase in level after HIIT and a decrease after ST in young men. Kouvelitoti et al. investigated the acute response in BTMs after interval treadmill running versus interval cycling (low mechanical load) in young women and found no differences in CTX-1 but an acute increase in sclerostin immediately after both exercise modes, which contrasts with the findings in young women in the current study (52). Acute physical activity is postulated to inhibit sclerostin (53), and plyometric exercise has been found to cause an immediate and transient increase in serum sclerostin levels in men, but not in prepubertal boys and girls (54). We speculate that the high load and rapid execution (i.e., high rate of force development) in our ST model could be the decisive factor regarding the sclerostin response, as this type of execution is more similar to plyometric exercise. Still, sclerostin’s response to exercise is not yet clear and requires further research, as conflicting reports exist regarding circulating sclerostin alterations, from elevation, no effect, and suppression (55).

In the elderly men, we observed a significant difference in LCN2_c_ levels between the two exercise modes, as it was increased immediately and 3 h after HIIT before returning to baseline values but was increased at 24 h only after ST. Few studies have explored the effects of exercise on circulating LCN2 in humans, and since it is regarded as a mechanosensor (29), we find it interesting that the acute effects of the two exercise modes differ in the current study, even though in elderly men only. The increase in LCN2_c_ after HIIT in the elderly men corresponds to the results from Ponzetti et al. describing increased serum LCN2 immediately after acute high-intensity exercise in young and middle-aged male runners (31), suggesting that HIIT is a potent stimulus for LCN2 response. In the young male participants, we also found an acute simple time effect on LCN2_c_, which increased immediately after HIIT and then decreased after 24 h, but these effects were not significantly different from ST.

### Effect of Participants’ Fitness Level and Acute Responses of Exercise

The participants included in this study all exhibited higher aerobic capacity, measured by V̇O_2max_ (between 8% and 18% above the average) for their sex and age compared to reference data from healthy Norwegian men and women aged 20–90 (56). The participants’ fitness level might modulate BTMs and related bone marker responses to acute training. Mieszkowski et al. described that serum CTX-1 increased after a Wingate high-intensity anaerobic test in physically active men, but not in professional gymnastics athletes (44), while Scott et al. found no significant differences in β-CTX response between endurance-trained and recreationally active men after exhaustive running (57). Interestingly, we found no association between 1RM or V̇O_2max_ and changes in plasma volume-corrected BTMs and related markers among the male participants, indicating that in the current setting, neither muscle strength nor cardiovascular fitness affected the training-induced changes in BTMs and related bone markers, in young and elderly men. In young women, muscle strength (1RM) was correlated with the significant main effect of time changes in all measured BTMs and related markers, but no correlations were detected with V̇O_2max_. The baseline levels of BTMs and related bone markers in the young women showed some variations between the two exercise modes’ baseline levels, which could not be detected among the male participants. Circulating levels of CTX-1 might vary during the menstrual cycle (58), and the use of oral contraceptives is known to decrease bone turnover (59), which partly can explain the observed variations in baseline values in the female group. We did not collect data on the menstrual cycle in the current study, but ten of the 19 women included reported the use of oral contraceptives. When analyzing the baseline levels of BTMs between users/non-users of oral contraceptives, we found no significant differences, possibly due to the relatively small sample size.

### Implications for Long-Term Training Adaptations

We do not know how acute and transient changes to individual BTMs and related markers translate in the long-term regarding changes in BMD and microarchitecture and, ultimately, to bone strength and future fracture risk. A recent systematic review of the effects of acute exercise on BTMs in middle-aged and older adults concluded that acute exercise is an effective tool to modify BTMs, but the response appears to be dependent on exercise modality, intensity, age, and sex (19). The current study indicates that both high-intensity running exercise and lower-extremity ST can provide favorable effects on bone metabolism, at least in younger individuals, which agrees with our previous findings of improved skeletal properties following 12 weeks of hack squat MST (11–13). Further research is needed to unravel whether this also applies to treadmill HIIT. The observed acute effects in BTMs and related biomarkers returned to baseline levels after 24 h, indicating that persistent training-induced changes from an intervention should be measured at later time points.

## Conclusion

Despite being inherently different in skeletal loading characteristics, HIIT and ST induced similar effects on bone metabolism markers in young adults of both sexes. The two exercise modes did not diverge in BTM and OC_c_ responses but did for sclerostin_c_ in young men and LCN2_c_ in elderly men. Baseline fitness measures were not correlated to changes in BTMs and related bone markers in the male groups, while muscle strength measured by 1RM was associated with changes in BTMs and related markers in the young female group.

Collectively, these findings demonstrate that beneficial skeletal effects on bone metabolism can be attained through both aerobic endurance and resistance exercise, although this effect seems to be attenuated with age. Mostly, these effects were blunted after 24 h, suggesting that persistent alterations following prolonged exercise interventions should be assessed at later time points.

## Study Strengths and Limitations

Our study has several strengths. We included participants across sex and age. All baseline and 24 h after exercise blood samples were taken in the morning, after overnight fasting, to rule out diurnal variations. The energy intake was controlled and similar for all participants during exercise sessions, and sessions were supervised in a laboratory setting with close individual monitoring by qualified staff. All analyses of serum markers were performed with concentrations corrected for potential ΔPV, using changes in total calcium as a hemoconcentration biomarker. All participants were thoroughly tested regarding their muscular strength and cardiovascular fitness. The current study also has some limitations. We did not measure BMD in our participants, so we cannot rule out that the bone status by means of BMD could affect our results.

The baseline and 24-h blood samples were drawn in the morning after overnight fasting. Participants received equal amounts of carbohydrate energy gel before performing the exercise, and it cannot be ruled out that the serum BTMs measured at post-test and 3 h after exercise samples might have been affected by nutrition intake. Studies have demonstrated that intake of carbohydrates causes a decrease in serum BTMs, especially CTX-1, for up to 2 h after intake (60–62). However, Sale et al. suggested that even though carbohydrate feeding during exercise reduces overall bone turnover in the hours following exercise, the balance between resorption and formation markers is maintained (63). A recent review stated circadian variation for BTMs CTX-1 and OC, with nighttime or early morning peak, but with less amplitude for P1NP and none for sclerostin (64). We tried to minimize the effects of circadian variation for BTMs by drawing blood at the same time of the day for all participants.

We measured serum tOC and were therefore unable to reveal a possible shift between the two forms of circulating OC (bone mineralization related carbonylated (cOC) and undercarboxylated (ucOC)). The optimal time for blood sampling for assessment of BTMs and related bone markers after training is not established. Ideally, blood tests should be taken every 30 min post-exercise for an extended period. For the feasibility of the study, we chose four time points for blood sampling (baseline, immediately after, 3, and 24 h after the exercise sessions). We cannot, however, rule out that we have “missed” the participants’ individual peak responses in BTMs, OC, sclerostin, and LCN2 after exercise. Also, for the feasibility of the study and to minimize the number of visits for the participants, the order of the exercise sessions was not randomized. Preferably, both sexes should have been evaluated also in the elderly group, but we chose to prioritize men in an attempt to undermine the estrogen component related to aging. In the context of skeletal health, it could be argued that elderly women would be more relevant, although previous studies suggest that comparable results as in the present study can be expected (33, 65).

## Data Availability Statement

The original contributions presented in the study are included in the article/supplementary material. Further inquiries can be directed to the corresponding author.

## Ethics Statement

The studies involving human participants were reviewed and approved by the Norwegian Regional Ethics Committee for Medical and Health Research (REK2018/926). The patients/participants provided their written informed consent to participate in this study.

## Author Contributions

Conceptualization: MM, US, and EW. Data collection and curation: CB, NA, MB, MM, and EW. Analysis: MS and AS. Formal analyses, statistics, and visualization: AS. Funding acquisition: MM, US, and AS. Project administration: MM. Supervision: MM and EW. Validation: MM and AS. Writing—original draft: AS. Writing—review and editing: all authors.

## Funding

AS, MS, US, and MM received funding from The Liaison Committee for education, research, and innovation in Central Norway.

## Conflict of Interest

The authors declare that the research was conducted in the absence of any commercial or financial relationships that could be construed as a potential conflict of interest.

## Publisher’s Note

All claims expressed in this article are solely those of the authors and do not necessarily represent those of their affiliated organizations, or those of the publisher, the editors and the reviewers. Any product that may be evaluated in this article, or claim that may be made by its manufacturer, is not guaranteed or endorsed by the publisher.
